# Down syndrome and postoperative hemodynamics in patients undergoing surgery for congenital cardiac communications

**DOI:** 10.1038/s41598-024-67097-4

**Published:** 2024-07-18

**Authors:** Eloisa Sassá Carvalho, Juliano Gomes Penha, Nair Yukie Maeda, Kelly Cristina O. Abud, Maria Francilene S. Souza, Claudia R. P. Castro, Johnny X. dos Santos, Juliana Pereira, Antonio Augusto Lopes

**Affiliations:** 1 Heart Institute (InCor), University of São Paulo School of Medicine, São Paulo, Brazil; 2 Pró-Sangue Foundation, São Paulo, Brazil; 3 Laboratory of Medical Investigation On Pathogenesis and Targeted Therapy in Onco-Immuno-Hematology (LIM-31), University of São Paulo, São Paulo, Brazil; 4 Department of Pediatric Cardiology and Adult Congenital Heart Disease, Heart Institute (InCor) – HCFMUSP, Av. Dr. Eneas de Carvalho Aguiar, 44, São Paulo, 05403-000 Brazil

**Keywords:** Congenital heart diseases, Pediatric cardiac surgery, Postoperative intensive care, Down syndrome, Pulmonary hypertension, Inflammatory mediators, Congenital heart defects, Cardiovascular biology

## Abstract

Although Down syndrome (DS) is considered a risk factor for hemodynamic instabilities (mainly pulmonary hypertension–PH) following surgery for congenital cardiac communications, many DS patients do surprising well postoperatively. We prospectively analyzed perioperative factors for a possible correlation with post-cardiopulmonary bypass (CPB) inflammatory reaction and postoperative PH in pediatric subjects. Sixty patients were enrolled (age 3 to 35 months), 39 of them with DS. Clinical and echocardiographic parameters (anatomical and hemodynamic) were computed preoperatively. Pulmonary and systemic mean arterial pressures (PAP and SAP) were assessed invasively intra and postoperatively. Immediate postoperative PAP/SAP ratio (PAP/SAP_IPO_) and the behavior of pressure curves were selected as primary outcome. Serum levels of 36 inflammatory proteins were measured by chemiluminescence preoperatively and 4 h post CPB. Of all factors analyzed, peripheral oxygen saturation (O_2_Sat, bedside assessment) was the only preoperative predictor of PAP/SAP_IPO_ at multivariate analysis (p = 0.007). Respective values in non-DS, DS/O_2_Sat ≥ 95% and DS/O_2_Sat < 95% subgroups were 0.34 (0.017), 0.40 (0.027) and 0.45 (0.026), mean (SE), p = 0.004. The difference between non-DS and DS groups regarding postoperative PAP curves (upward shift in DS patients, p = 0.015) became nonsignificant (p = 0.114) after adjustment for preoperative O_2_Sat. Post-CPB levels of at least 5 cytokines were higher in patients with O_2_Sat < 95% *versus* those at or above this level, even within the DS group (p < 0.05). Thus, a baseline O_2_Sat < 95% representing pathophysiological phenomena in the airways and the distal lung, rather than DS in a broad sense, seems to be associated with post-CPB inflammation and postoperative PH in these patients.

## Introduction

Pulmonary vascular abnormalities pose a risk for hemodynamic disturbances following surgical repair of congenital cardiac septal defects. Although pulmonary vascular disease associated with congenital heart disease is decreasing in countries with privileged referral patterns, in most of the world it continues to be a major cause of postoperative complications and residual pulmonary hypertension late after operation. Severe postoperative hemodynamic disturbances initiated with or aggravated by pulmonary vascular tone instabilities are associated with increased length of intensive care unit stay and hospitalization, and high mortality rates. Late referral, the presence of genetic abnormalities and extracardiac syndromes, pediatric comorbidities and the complexity of the cardiac anomaly have been considered classical risk factors for pulmonary hypertension and postoperative complications^1,2^. In patients suspected to have high pulmonary vascular resistance, operability and the potential for reversibility of pulmonary vascular lesions are generally assessed by preoperative cardiac catheterization with pulmonary vasodilator testing^3,4^. While right heart catheterization is the gold standard procedure to decide about surgery in selected cases, most patients with unrestrictive cardiac communications are assigned to operation based on noninvasive diagnostic evaluation. Furthermore, patients who are deemed operable based on a positive pulmonary vasodilator test are not totally free from postoperative pulmonary vascular disturbances since a positive test actually characterizes a dynamic pulmonary circulation which may become reactive under certain stimuli.

Down syndrome (trisomy 21) is the most common chromosomal abnormality, affecting approximately 1 in 700 births. Although data on the occurrence of babies born with Down syndrome in Brazil are unclear, an incidence of 4 in 10,000 live births was recently reported^5^. It is associated with a number of comorbidities and has been considered a major risk factor for early development of pulmonary vascular disease and pulmonary hypertension, particularly in subjects with congenital heart disease. Several conditions contribute to the abnormal behavior of the pulmonary circulation in individuals with Down syndrome including abnormal lung development and angiogenesis, upper and lower airway obstructions, bronchopulmonary dysplasia, recurrent pneumonia, obstructive sleep apnea, increased hemodynamic stress in patients with congenital cardiac shunts and endothelial dysfunction with a shift toward a proinflammatory phenotype^6^. All these conditions may cause changes in systemic oxygen saturation as a result of pulmonary ventilation-perfusion mismatch and/or right-to-left intracardiac shunting.

Of particular concern in regard to the development of pulmonary vascular abnormalities is the observation that Down syndrome patients have increased systemic inflammation and altered immune response. For example, four interferon receptors are encoded on human chromosome 21 and are highly expressed in syndromic individuals in comparison with disomic controls^7^. Interferon is involved in inhibition of nitric oxide synthase, up-regulation of endothelin 1 and induction of chemokine IP-10 (interferon-inducible protein 10) which is central to pulmonary vascular smooth muscle cell growth^8–10^. However, controversy remains whether the presence of congenital cardiac anomalies itself has impact on the levels of inflammatory mediators in these subjects^11^. Also, it is not known if Down syndrome patients have any particular behavior in terms of post-cardiopulmonary bypass systemic inflammatory reaction with potential impact on postoperative hemodynamics.

Considering that many Down syndrome patients with unrestrictive cardiac communications do surprisingly well after surgery^12^, we designed the present study as an attempt to identify preoperative and intraoperative factors that might differentiate between subjects with stable *versus* unstable clinical course. We also investigated whether Down syndrome patients or any particular subset of syndromic individuals had differential responses in terms of postoperative inflammatory reaction.

## Methods

### Patients

This was a prospective cohort study comprising patients who were referred to the Heart Institute (InCor), University of São Paulo School of Medicine, São Paulo, Brazil from November 2016 to September 2021, for surgical repair of congenital cardiac communications. Patients entered the study consecutively if they met the following inclusion criteria: age 1 month to 3 years; biventricular cardiac physiology; presence of at least one unrestrictive post-tricuspid cardiac communication (diameter of the communication greater than 50% of the aortic annulus diameter) without pulmonary stenosis; and clinical and echocardiographic signs of at least moderately elevated pulmonary arterial pressure (preferably with a systolic pulmonary arterial pressure > 40 mmHg whenever parameter obtainment was possible); The exclusion criteria were: presence of extracardiac syndromes other than Down syndrome; complex cardiac anomalies, including those anatomically or physiologically characterized as univentricular hearts; predominant right-to-left intracardiac shunting, suggesting high pulmonary vascular resistance associated with advanced pulmonary vasculopathy; presence of any signs of ongoing or recent inflammatory or infectious diseases; and reoperation. The Heart Institute is a tertiary referral center. A great percentage of its pediatric patient population is represented by neonates, subjects with complex cardiac anomalies and candidates to reoperation. In this way, although this study was initially planned to include patients aged 1 month to 2 years, we needed to consider an extended age range (up to 3 years) in order to have an adequately sized study population. Forty-seven healthy pediatric subjects (including 24 nonsyndromic subjects aged 1 to 27 months) entered the study as controls for the laboratory determinations of inflammatory mediators. This group was also made up of individuals with Down syndrome (23 subjects aged 1 to 35 months). Nonsyndromic and syndromic individuals included for this specific purpose were from the same geographic area as that of the patients. They did not have any relevant hemodynamic abnormalities or signs of pulmonary hypertension upon evaluation by the Heart Institute team. Data from the control group were used only for descriptive purposes. They were not included in inferential analysis. All participants were included after obtainment of a written informed consent from their parents. All procedures of the study were approved by the ethics committee of the Heart Institute, University of São Paulo School of Medicine, São Paulo, Brazil (approval number: 2.068.696). The authors also confirm that the entire research was performed in accordance with the Declaration of Helsinki.

### Preoperative evaluation and perioperative management

Diagnostic evaluation consisted of a detailed clinical history, physical examination, chest radiographs, electrocardiogram and a transthoracic echocardiogram. The diagnosis of Down syndrome was based on phenotypic features and was confirmed by genetic testing. Patients were evaluated for the presence or absence of clinical features suggestive of pulmonary overcirculation: dyspnea, congestive heart failure and failure to thrive. Transthoracic echocardiography was used to assess cardiovascular anatomy and hemodynamic parameters. Systolic pulmonary arterial pressure and mean pulmonary arterial pressure were estimated in patients for whom reliable curves of tricuspid and pulmonary regurgitation jets (respectively) could be obtained. Some patients presented with a pulmonary/systemic blood flow ratio < 2.00 and peripheral oxygen saturation < 95%, thus requiring special attention. However, no patients had predominant right-to-left flow across the septal defect and/or oxygen saturation < 85% (exclusion criteria). The need for preoperative cardiac catheterization was established based on previously reported consensus^13^.

Pulmonary and systemic arterial pressures were measured directly in the operating room, before and after cardiopulmonary bypass. In our institution, patients who are at risk for postoperative hemodynamic instabilities, i.e., those with unrestrictive cardiac communications and preoperative signs of pulmonary hypertension are routinely weaned from cardiopulmonary bypass on 20 ppm inhaled nitric oxide and have a pulmonary arterial catheter inserted by the surgeon to facilitate hemodynamic monitoring in the intensive care unit. Postoperative analgosedation was performed using fentanyl, midazolan and ketamine singly or in combination. Alternatively, morphine and dexmetomidine were used in combination in patients with a very stable clinical course. Milrinone, epinephrine and norepinephrine were administered as inotropic/vasoactive agents. The total amount of drugs used was computed using the vasoactive-inotropic score^14^. Patients were kept on inhaled nitric oxide during the entire period of mechanical ventilation, except those in whom ventilation was prolonged for reasons other than pulmonary hypertension.

### Postoperative hemodynamics as primary outcome

Hemodynamic parameters were analyzed in two ways. Initially, immediate postoperative pulmonary/systemic mean arterial pressure ratio (PAP/SAP_IPO_, one value per patient) was calculated as the mean of first 4 values obtained in the intensive care unit (readings taken at 2 h intervals). In a previous study of ours, a PAP/SAP_IPO_ > 0.40 was predictive of unstable clinical course^15^. Subsequently, curves were constructed for pulmonary and systemic arterial pressures and peripheral oxygen saturation, and were comparatively analyzed in patients with and without Down syndrome. Although parameters were recorded for about 2.5 days in the majority of patients, for the specific purpose of comparative analysis we decided to consider data obtained during the first 12 h of intensive care unit stay. In that period, patients were still deeply sedated, clinically stable and already free from major post-cardiopulmonary bypass hemodynamic instabilities.

### Inflammatory markers

Peripheral venous blood was collected 3 days before surgery and 4 h after cardiopulmonary bypass termination for analysis of serum levels of 36 inflammatory mediators: complement component 5/5a (C5/C5a); CD40 ligand (CD40L); granulocyte colony-stimulating factor (G-CSF); granulocyte–macrophage colony-stimulating factor (GM-CSF); growth-regulated oncogene alpha (GROα); human CC chemokine I-309 (I-309); intercellular adhesion molecule-1 (ICAM-1); interferon gamma (IFN-γ); interleukins (IL) 1 alpha, 1 beta, 2, 4, 5, 6, 8, 10, 12, 13, 16, 17, 17E, 18, 21, 27 and 32 alpha; interleukin-1 receptor antagonist (IL-1RA); interferon gamma-induced protein-10 (IP-10); interferon-inducible T cell alpha chemoattractant (I-TAC); monocyte chemoattractant protein-1 (MCP-1); macrophage migration inhibitory factor (MIF); macrophage inflammatory protein-1 alpha/beta (MIP-1α/β); regulated on activation, normal T cell expressed and secreted (RANTES); plasminogen activator inhibitor-1 (Serpin E1); stromal cell-derived factor-1 (SDF-1); tumor necrosis factor alpha (TNF-α); and soluble triggering receptor expressed on myeloid cells-1 (sTREM-1). Proteins were analyzed by immunoblotting and semiquantified by chemiluminescence (Human Cytokine Array, R&D Systems, Minneapolis, MN, USA). Kits containing 4 membranes with immobilized antibodies against 36 proteins were used to analyze pre- and postoperative samples in the same assay (2 patients per kit). The results were expressed as the average signal of each pair of duplicate spots (units of pixel intensity, upi). In order to avoid excessive variations of results, all samples were stored at −80 °C and processed at the end of the study. Besides, internal standards provided by the kit manufacturer were used to adjust for small variations between the kits.

### Postoperative events as secondary outcome

We defined cardiovascular (hemodynamic) and respiratory events beyond the concept of pulmonary hypertensive crises, encompassing the following instabilities: 1, typical pulmonary hypertensive crises defined as sustained elevation of pulmonary arterial pressure (pulmonary/systemic mean arterial pressure ratio > 0.75) with a decline in systemic pressure (≥ 20%) and peripheral oxygen saturation (< 90%)^16^; 2, systemic hypotension with a pulmonary/systemic mean arterial pressure ratio in the range of 0.50–0.75 requiring frequent changes in the doses of vasoactive drugs; 3, prolonged and/or recurrent hemodynamic and respiratory disturbances not reverted by sedation and manual ventilation; and 4, all critical instabilities requiring cardiorespiratory resuscitation. Elevations of pulmonary arterial pressure, even to suprasystemic levels, that were rapidly reverted by sedation and manual ventilation were not characterized as events. Interpretations were made independently by 3 observes (ESC and AAL, co-authors, and on-duty intensivist).

### Data obtainment

The professional who was responsible for the laboratory determinations of inflammatory markers (NYM, co-author) had no access to any preoperative or postoperative data throughout the study. Thus, clinical and laboratory data were obtained and computed in a blinded fashion.

### Statistical analysis

The sample size was calculated to demonstrate statistically significant differences in the parameter PAP/SAP_IPO_ between the study groups. In order to compare 2 groups (i.e., Down-syndrome *versus* non-Down syndrome patients), a total of 50 patients was considered sufficient to demonstrate a standardized difference of approximately 1.05 SD (power 95.4%, significance level 0.05). Considering the possibility of subgroup analysis (e.g., comparison between 3 subgroups), a total of 57 individuals was considered adequate to demonstrate standardized differences of approximately 1.04 SD of residuals (power 81.3%, significance level 0.05). The study was designed to include 60 patients. The sample size and power were calculated using the software G*Power, version 3.1.

Unless otherwise specified, numeric variables are presented as medians with interquartile ranges. Categorical variables are presented as number of cases and percentages. In descriptive analyses, comparisons between groups were performed using the Mann–Whitney test and Kruskal–Wallis test. Differences involving categorical variables were tested using the Chi-square family of tests. The Wilcoxon test and Pearson’s coefficient of correlation were used to test for differences and associations within subjects. Inferential statistics was used to analyze outcomes and their predictors. All dependent variables were tested for closeness to the normal (Gaussian) distribution. In most instances, closeness to the normal distribution was obtained using the Box-Cox transformation of dependent variables. Hemodynamic outcomes were analyzed in patient groups using the general linear model (one-way GLM analysis or two-way GLM analysis for repeated measures). Predictors of postoperative events (categorical outcome) were analyzed using logistic regression analysis. In view of the complex distributions observed for most biological markers (inflammatory proteins), we opted to compare groups using nonparametric statistics (Mann–Whitney test). In all tests, 0.05 was set as the significance level. In all parametric and nonparametric procedures aimed at comparing more than two groups, significance values were adjusted using the Bonferroni correction for multiple tests. Statistical analysis was performed using the SPSS statistical software, version 28 (IBM, Armonk, NY, USA).

### Ethical approval and consent to participate

The study protocol was approved by the Institutional Scientific and Ethics Committee, Heart Institute, University of São Paulo School of Medicine, São Paulo, Brazil, approval N^o^. 2.068.696. Informed consent was obtained from the patient’s parents before enrollment in the study.

## Results

Sixty patients were enrolled with age range of 3 to 35 months. Thirty-nine of them had Down syndrome. Demographic and diagnostic data of syndromic and nonsyndromic individuals are shown in Table 1. All but one patient had unrestrictive ventricular septal defect or complete atrioventricular septal defect either isolated or associated with atrial septal defect (secundum type) or patent ductus arteriosus. In the present cohort, atrioventricular septal defects were present in syndromic individuals only. All but one patient presenting with peripheral oxygen saturation < 95% were syndromic individuals with atrioventricular septal defect (n = 19). However, there were patients with Down syndrome presenting with normal oxygen saturation as well (n = 20, 5 with atrioventricular canal). Syndromic patients had lower levels of systemic arterial pressure compared to nonsyndromic ones. Seven patients underwent preoperative cardiac catheterization. All of them were Down syndrome individuals presenting with no evident signs of congestive failure. Peripheral oxygen saturation in this subgroup was 90% (89–96%) (median with interquartile range) compared with 96% (94–98%) in patients not requiring cardiac catheterization (p = 0.030). Despite that, pulmonary vascular resistance was found to be only mildly elevated (3.8 [3.0–4.9] Wood units x m^2^) with a decrease following nitric oxide inhalation (3.4 [1.5–4.3] Wood units x m^2^, p = 0.043), thus allowing for safe assignment to cardiac surgery.

**Table 1:** Demographic and diagnostic data in patient groups.

	Down syndrome		p value
	Absent (n = 21)	Present (n = 39)	
Age, months	12 (7–17)	11 (8–15)	0.840
Gender, M:F	10:11	12:27	0.312
Weight, Kg	7.3 (5.4–8.3)	6.5 (5.8–7.1)	0.636
Height, cm	70 (63–75)	65 (62–69)	0.195
Cardiac anomaly, n (%)			
Ventricular septal defect	21 (100.0)	14 (35.9)	
Atrioventricular septal defect	0 (0.0)	24 (61.5)	< 0.001
Aortopulmonary window	0 (0.0)	1 (2.6)	
Clinical presentation, n (%)*			
A	21 (100.0)	33 (84.6)	0.082
B	0 (0.0)	6 (15.4)	
Peripheral oxygen saturation, %	97 (96–99)	96 (92–97)	0.002
Echocardiographic data			
Systolic pulmonary arterial pressure, mmHg†	70 (60–80)	76 (67–85)	0.494
Mean pulmonary arterial pressure, mmHg‡	49 (34–58)	43 (33–51)	0.280
Pulmonary/systemic blood flow ratio	2.50 (1.80–3.35)	2.10 (1.70–2.90)	0.267
Velocity–time integral of blood flow in pulmonary veins, cm§	22.0 (20.0–24.7)	21.1 (19.0–25.3)	0.659
TAPSE, mmǁ	14 (12–19)	15 (13–17)	0.640
Pre-cardiopulmonary bypass intraoperative data			
Mean pulmonary arterial pressure, mmHg	32 (27–43)	32 (28–37)	0.576
Mean systemic arterial pressure, mmHg	50 (42–57)	41 (38–49)	0.007
Pulmonary/systemic mean arterial pressure ratio	0.70 (0.62–0.85)	0.80 (0.63–0.89)	0.204

Numeric variables are presented as medians with interquartile ranges. Differences were tested using the Mann–Whitney test. For categorical variables, differences were analyzed using the Chi-square family of tests.

*A, presence of clinical features suggestive of pulmonary overcirculation (dyspnea, congestive heart failure, and failure to thrive); B, absence of such features, thus suggesting elevation of pulmonary vascular resistance.

^†^Due to technical limitations, reliable data were obtained in 28 syndromic and 6 non syndromic patients.

^‡^Reliable data were available from 12 syndromic and 16 nonsyndromic individuals.

^§^Values < 20 cm indicate absence of pulmonary overcirculation, and are generally associated with heightened pulmonary vascular resistance in pediatric patients with unrestrictive cardiac communications.

ǁTricuspid annular plane systolic excursion is a parameter directly related to right ventricular systolic function.

All patients underwent complete surgical repair of cardiac anomalies. Cardiopulmonary bypass time in patients with ventricular septal defect and those with atrioventricular septal defect was 102 min (85–130 min) and 152 min (136–166 min), respectively. A 10-min ultrafiltration procedure was performed at the end of cardiopulmonary bypass in all patients. Pre- and post-cardiopulmonary bypass hemodynamic measurements showed the following changes: a decrease in mean pulmonary arterial pressure from 32 (27–43) mmHg to 21 (19–24) mmHg (p < 0.001) and 32 (28–37) mmHg to 22 (18–27) mmHg (p < 0.001) in nonsyndromic and syndromic patients, respectively; an increase in mean systemic arterial pressure from 50 (42–57) mmHg to 52 (49–60) mmHg (p = 0.050) and 41 (38–49) mmHg to 52 (49–58) mmHg (p < 0.001), respectively; and a decrease in pulmonary/systemic mean arterial pressure ratio from 0.70 (0.62–0.85) to 0.36 (0.32–0.44) (p < 0.001) and 0.80 (0.63–0.89) to 0.42 (0.33–0.49) (p < 0.001), respectively.

Postoperative hemodynamics was analyzed first by obtaining the parameter PAP/SAP_IPO_. In the entire patient population, PAP/SAP_IPO_ ranged from 0.19 to 0.74 (0.37 [0.32–0.46]). Greater values were seen in patients with Down syndrome compared to nonsyndromic individuals (respectively, 0.40 [0.33–0.49] and 0.34 [0.29–0.42], p = 0.005). Values were even greater in the subgroup of patients requiring preoperative cardiac catheterization (0.54 [0.47–0.62] compared with 0.36 [0.32–0.44] in subjects not requiring catheterization, p = 0.002). Subsequent analysis of preoperative and intraoperative variables to identify possible associations with PAP/SAP_IPO_ showed several potential predictors in addition to Down syndrome (Table 2). However, only preoperative peripheral oxygen saturation measured at bedside and pre-cardiopulmonary bypass PAP/SAP ratio computed in the operating room remained in the final multivariate statistical model (Table 2).

**Table 2:** Factors influencing early postoperative hemodynamics*.

	Univariate analysis		Multivariate analysis 1†		Multivariate analysis 2‡	
	Coefficient	p value	Coefficient	p value	Coefficient	p value
Age, months	−0.005	0.305	–	–	–	–
Gender (female)	+0.029	0.445	–	–	–	–
Down syndrome (present)	+0.104	0.005	+0.057	0.101	–	–
Weight, Kg	−0.028	0.175	–	–	–	–
Cardiac anomaly (AVSD)	+0.089	0.014	–	–	–	–
Peripheral oxygen saturation, %	− 0.038	< 0.001	−0.021	0.052	−0.028	0.007
Pulmonary/systemic blood flow ratio§	−0.023	0.595	–	–	–	–
Pulmonary venous flow (VTIPV), cmǁ	+0.012	0.231	–	–	–	–
TAPSE, mm**	−0.029	0.011	–	–	–	–
Pre-CPB PAP, mmHg	+0.011	0.010	–	–	–	–
Pre-CPB SAP, mmHg	−0.008	0.040	–	–	–	–
Pre-CPB PAP/SAP	+0.729	< 0.001	+0.554	0.002	+0.564	0.002
CPB duration	+0.003	0.006	–	–	–	–

*CPB* cardiopulmonary bypass, *PAP and SAP* respectively, mean pulmonary arterial pressure and mean systemic arterial pressure, *PAP/SAP* pulmonary/systemic mean arterial pressure ratio, *TAPSE* tricuspid annular plane systolic excursion.

*Defined as immediate postoperative pulmonary/systemic mean arterial pressure ratio (PAP/SAPIPO, mean of first 4 values, readings at 2 h intervals). Factors and covariates were analyzed using the General Linear Model after Box-Cox transformation of the dependent variable (PAP/SAPIPO).

^†^Stepwise procedure for variable selection using alpha of 0.15 to enter and to remove variables.

^‡^Same procedure using alpha of 0.10 to enter and to remove variables.

^§^ Transthoracic echocardiography.

ǁVelocity-time integral of blood flow in pulmonary veins (transthoracic echocardiography) is directly related to the magnitude of pulmonary blood flow.

**Tricuspid annular plane systolic excursion is a parameter directly related with right ventricular systolic function.

**Figure 1:**
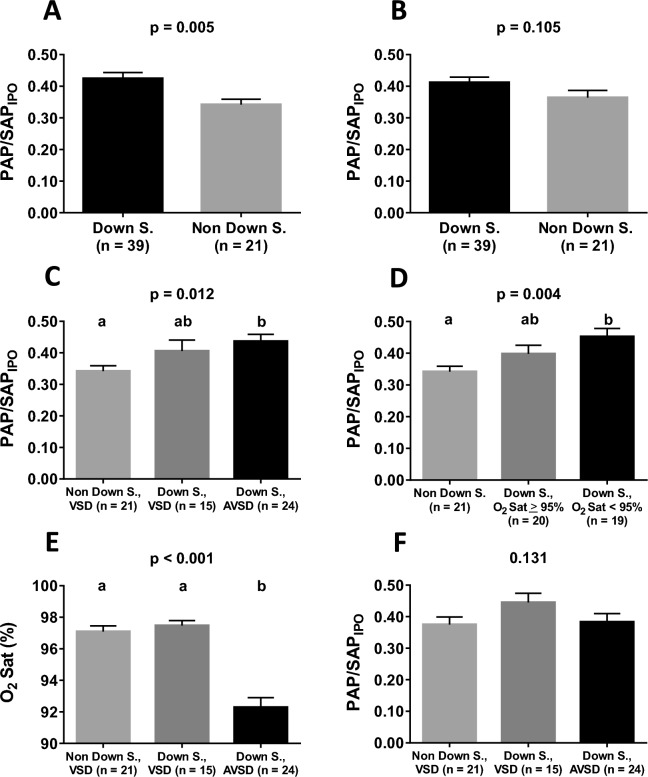
Role of Down syndrome, type of cardiac anomaly and preoperative peripheral oxygen saturation (O_2_ Sat) on early postoperative hemodynamics (PAP/SAP_IPO_, pulmonary/systemic mean arterial pressure ratio, mean of first four values computed in the intensive care unit, readings at 2 h intervals). Analyses were performed using the general linear model after Box-Cox transformation of the dependent variable. Results are presented as means with SE (**A**,C–**E**) or adjusted means with SE after inclusion of baseline oxygen saturation as a covariate in the statistical model (**B**,**F** with covariate p values of 0.006 and 0.005, respectively). In (**C**–**E**) groups not sharing the same letter were different (*post-hoc* multiple comparisons). *AVSD* atrioventricular septal defect, *VSD* ventricular septal defect.

The role of Down syndrome, type of cardiac anomaly and baseline oxygen saturation in predicting postoperative hemodynamics was further investigated using bivariate analysis as illustrated in Fig. 1. The difference between syndromic and nonsyndromic individuals with respect to PAP/SAP_IPO_ became statistically nonsignificant when means were adjusted for oxygen saturation (Fig. 1A,B). The greatest values of PAP/SAP_IPO_ were actually seen in Down syndrome patients presenting with peripheral oxygen saturation < 95% (Fig. 1D). Although the type of cardiac anomaly could be looked on as an important predictor (Fig. 1C), the difference between groups became nonsignificant when baseline oxygen saturation (lowest levels observed in subjects with atrioventricular septal defect, Fig. 1E) was taken into consideration (Fig. 1F). Thus, oxygen saturation was characterized as the most important preoperative predictor of postoperative hemodynamics. In patients with Down syndrome *versus* nonsyndromic ones, the risk of having a PAP/SAP_IPO_ > 0.40, which may be looked on as an important elevation of pulmonary artery pressure postoperatively, was 2.62 (0.85–8.20) (odds ratio with 95% CI p = 0.095). However, in patients with baseline oxygen saturation < 95% *versus* those who were at or above this level, the risk was 5.44 (1.69–17.57) (p = 0.005). Each 1% reduction in preoperative oxygen saturation was associated with an increase of 0.015 in PAP/SAP_IPO_ (p < 0.001).

**Figure 2:**
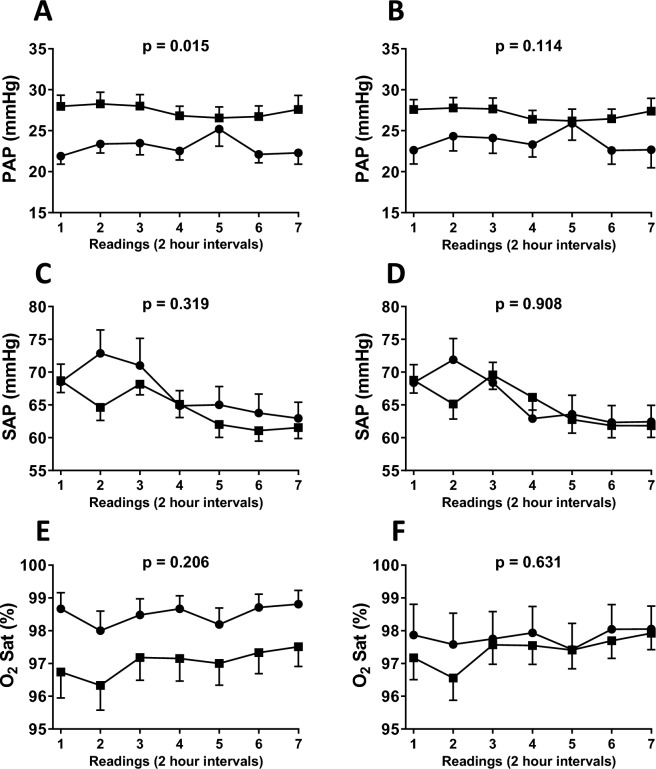
Mean pulmonary and systemic arterial pressure (respectively, PAP and SAP) and peripheral oxygen saturation (O_2_Sat) computed during the first 12 h of postoperative intensive care for patients with (squares, n = 39) and without (circles, n = 21) Down syndrome. Data were analyzed using the general linear model for repeated measures after Box-Cox transformation of the dependent variable. Results are presented as means with SE (**A**,**C**,**E**) or adjusted means with SE using baseline oxygen saturation as a covariate in the model (**B**, **D**,**F**, p < 0.05 for the covariate in all tests).

Hemodynamics was also analyzed by examining pulmonary and systemic arterial pressure curves. Figure 2 shows pressure and oxygen saturation curves for patients with and without Down syndrome. While pulmonary artery pressure remained relatively stable with higher levels in syndromic individuals (Fig. 2A), systemic arterial pressure tended to decline initially, with no difference between groups (Fig. 2C). Lower but not statistically different oxygen saturation levels were computed for Down syndrome patients (Fig. 2E). However, and again when curves were normalized by adjusting for baseline (preoperative) oxygen saturation, between-group differences became unimpressive for all three parameters (Fig. 2B,D,F).

Analysis of all 36 inflammatory markers, at baseline, showed differences between patients and controls only for complement components C5/C5a. Levels in controls, nonsyndromic patients, syndromic patients with oxygen saturation ≥ 95% and syndromic patients with oxygen saturation < 95% were 1776 (842–3954) upi, 2225 (1302–6005) upi, 2842 (810–6089) upi and 4644 (2456–8159) upi respectively, with a significant difference between the former group and the latter (p = 0.014). Levels in the latter group were also higher when compared to the subgroup of controls without Down syndrome (4644 [2456–8159] upi and 1820 [1144–4550] upi respectively, p = 0.009). For the entire patient population, there was a negative correlation between serum C5/C5a and bedside oxygen saturation (r = −0.37, p = 0.009). Analysis of inflammatory markers within the group of surgical patients showed no differences between syndromic and nonsyndromic individuals. Data analysis within the controls showed a single difference between syndromic and nonsyndromic subjects, with higher levels of ICAM-1 in the former group (respectively, 48105 [37169–52844] upi and 38765 [30290–43647] upi, p = 0.005).

Cardiopulmonary bypass was followed by changes in hematological parameters. Compared to baseline, there was a 67% decrease in lymphocyte count, a 46% increase in monocytes and a 2.3-fold increase in neutrophils with a 10.9-fold increase in neutrophil-to-lymphocyte ratio (p < 0.001 for all comparisons). Besides, there was a marked decrease in platelet count (58%, p < 0.001) with no immediate change in platelet volume. The systemic inflammatory reaction was also characterized by changes in serum levels of inflammatory proteins as shown in Table 3. While several proteins increased from baseline, others decreased (C5/C5a, CD40L, GRO alpha, Serpin-E1 and RANTES). The decrease in serum RANTES was directly correlated with the amount of vasoactive/inotropic drugs required to stabilize post-cardiopulmonary bypass hemodynamics (expressed as vasoactive-inotropic score, r = 0.43, p = 0.002), probably reflecting the reduction and functional exhaustion of T lymphocytes. Post-cardiopulmonary bypass level of CD40L was directly correlated with platelet count (r = 0.34, p = 0.019).

**Table 3:** Serum levels of inflammatory mediators at baseline and 4 h after cardiopulmonary bypass termination.

	Baseline	Post-CPB	p value
C5/C5a, upi	3193.0 (1199.0–6766.1)	1795.9 (1073.0–3577.8)	0.021
CD40L, upi	6148.3 (3129.9–10662.3)	2474.0 (1263.3–4974.8)	< 0.001
G-CSF, upi	447.6 (264.7–813.8)	1222.0 (684.5–1989.9)	< 0.001
GM-CSF, upi	327.2 (160.6–439.6)	278.7 (171.3–397.3)	0.753
GRO alpha, upi	1946.6 (1319.4–3249.5)	1301.1 (757.4–2134.5)	0.001
I-309, upi	504.5 (253.5–1157.5)	623.0 (300.1–935.1)	0.995
ICAM-1, upi	41303 (31628–52121)	39366 (28739–48650)	0.398
IFN-gamma, upi	293.6 (180.1–407.0)	285.0 (187.2–431.8)	0.302
IL-1 alpha, upi	364.6 (202.3–616.7)	366.0 (227.4–726.5)	0.624
IL-1 beta, upi	170.1 (123.8–470.2)	224.3 (121.8–435.6)	0.807
IL-1RA, upi	1626.2 (729.6–2450.3)	15023.1 (11423.7–20346.6)	< 0.001
IL-2, upi	192.3 (120.1–490.2)	222.3 (146.5–378.8)	0.666
IL-4, upi	336.8 (165.7–487.9)	295.0 (206.2–460.8)	0.369
IL-5, upi	126.3 (78.6–240.0)	136.2 (75.4–214.0)	0.630
IL-6, upi	300.7 (179.9–620.7)	1197.5 (562.7–2127.9)	< 0.001
IL-8, upi	266.9 (148.4–446.5)	481.5 (231.0–827.7)	< 0.001
IL-10, upi	250.0 (141.0–390.5)	283.9 (204.5–460.6)	0.050
IL-12p70, upi	261.2 (151.4–420.6)	239.1 (131.5–412.5)	0.554
IL-13, upi	619.1 (317.5–1208.2)	671.2 (355.9–1157.1)	0.130
IL-16, upi	591.5 (290.0–1085.4)	1858.7 (1170.8–2840.2)	< 0.001
IL-17, upi	293.2 (176.6–476.9)	273.3 (175.2–521.3)	0.225
IL-17E, upi	399.6 (207.6–638.9)	349.8 (239.3–602.4)	0.781
IL-27, upi	282.8 (156.5–415.1)	251.4 (158.4–406.4)	0.817
IL-32 alpha, upi	582.5 (281.3–976.9)	442.2 (284.3–940.4)	0.133
IP-10, upi	786.9 (424.8–1375.0)	1518.6 (787.0–3825.9)	< 0.001
I-TAC, upi	519.1 (278.9–912.8)	503.5 (319.0–807.3)	0.959
MCP-1, upi	439.3 (232.2–824.0)	675.8 (378.2–1028.0)	0.001
MIF, upi	5529.2 (4652.0–7003.4)	7609.0 (6733.4–9934.9)	< 0.001
MIP-1 alpha/beta, upi	524.4 (268.6–878.2)	490.6 (280.6–921.0)	0.635
Serpin E1, upi	44650 (34461–56664)	36631 (31107–47622)	0.006
RANTES, upi	61983 (47488–71255)	50689 (39347–65383)	0.004
SDF-1, upi	4348.2 (3333.3–6126.8)	4321.0 (2910.9–6629.9)	0.933
TNF-alpha, upi	305.8 (149.4–584.6)	303.9 (203.1–584.0)	0.243
sTREM-1, upi	280.0 (177.3–533.0)	284.7 (195.6–583.3)	0.462
IL-18, upi	1058.5 (544.0–1576.3)	1055.0 (557.8 -1798.6)	0.103
IL-21, upi	411.1 (265.7–731.6)	414.4 (282.2–684.3)	0.440

Levels of inflammatory proteins were determined as units of pixel intensity (upi, chemiluninescence) and are presented as medians with interquartile ranges. Differences were analyzed using the Wilcoxon test.

**Figure 3:**
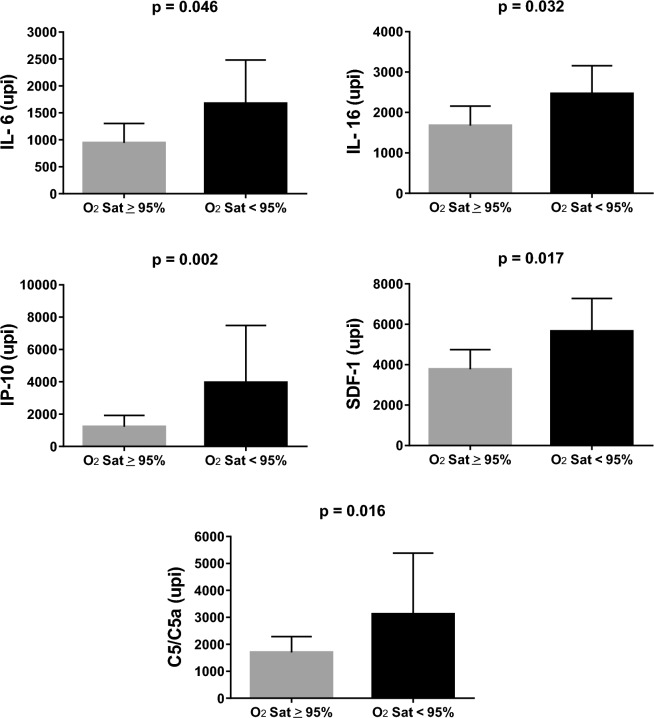
Serum levels of inflammatory mediators 4 h after weaning from cardiopulmonary bypass in patients with preoperative peripheral oxygen saturation (O_2_Sat) < 95% (n = 20) compared to those who were at or above this level (n = 40). Shown are the levels of interleukins 6 and 16 (IL-6 and IL-16), interferon gamma-induced protein 10 (IP-10), stromal cell-derived factor 1 (SDF-1) and complement components 5/5a (C5/C5a). Protein levels were determined as units of pixel intensity (upi) and results are presented as geometric means with 95% CI. The Mann–Whitney test was used for all comparisons.

**Figure 4:**
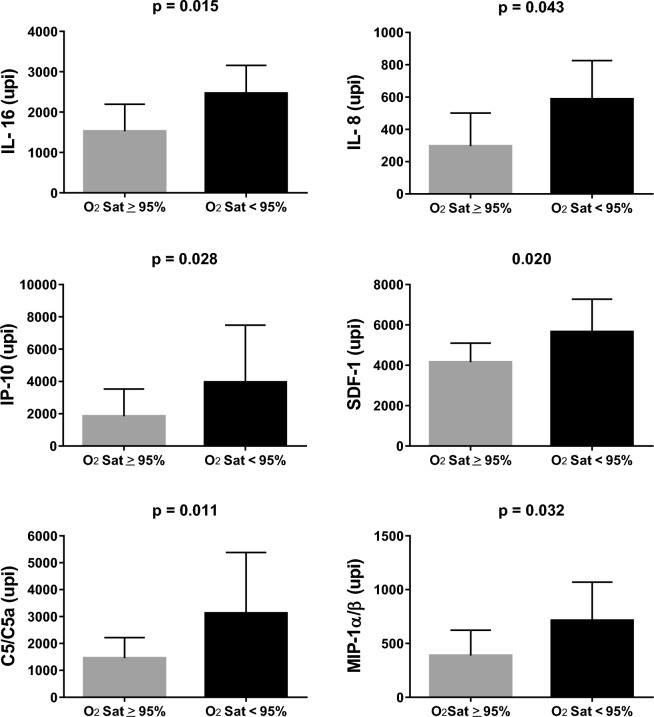
Post-cardiopulmonary bypass serum levels of inflammatory proteins for the specific group of patients with Down syndrome. Comparisons were made between patients with preoperative peripheral oxygen saturation (O_2_Sat) < 95% (n = 19) and those who were at or above this level (n = 20). Shown are the levels of interleukins 8 and 16 (IL-8 and IL-16), interferon gamma-induced protein 10 (IP-10), stromal cell-derived factor 1 (SDF-1), complement components 5/5a (C5/C5a) and macrophage inflammatory protein 1 alpha and beta (MIP-1alpha and MIP-1beta). Results are presented as geometric means with 95% CI. Differences were tested using the Mann–Whitney test.

At preliminary analysis, Down syndrome patients seemed to have higher postoperative levels of inflammatory proteins compared to nonsyndromic ones. Respective levels for IL-6 were 1791 (836–2755) upi and 1010 (368–1288) upi (p = 0.017); for IP-10, levels were 2353 (1010–5878) upi and 936 (362–1574) upi (p = 0.004). However, subsequent analyses showed that the magnitude of post-cardiopulmonary bypass inflammatory reaction was in fact more closely related to baseline oxygen saturation as illustrated in Figs. 3, 4. Considering the 13 proteins whose levels changed postoperatively relative to baseline (Table 3), significant correlations between postoperative levels and cardiopulmonary bypass duration was observed only for IL-6 (r_s_ = 0.32, p = 0.033), IP-10 (r_s_ = 0.35, p = 0.020) and IL-1RA (r_s_ = 0.29, p = 0.038).

During the intensive care unit stay, 14 patients had clinical events characterized by interrelated hemodynamic and respiratory instabilities as defined. The occurrence of such instabilities had a direct impact on the duration of mechanical ventilation (p < 0.001). Postoperative events could not be predicted on the basis of any preoperative parameters. In particular, events occurred in 9 Down syndrome patients (23%) and 5 nonsyndromic individuals (24%) (p = 0.999). However, events could be effectively predicted based on post-cardiopulmonary bypass mean systemic arterial pressure measured in the operating room, which was characterized as a protective factor (odds ratio 0.46 for quartiles, 95% CI 0.24–0.89, p = 0.021) and PAP/SAP_IPO_, identified as a risk factor (odds ratio 2.62 for quartiles, 95% CI 1.32–5.20, p = 0.006). Thus, preoperative oxygen saturation played and indirect role in determining the risk of postoperative events. One patient died of rapid-onset systemic hypotension and bradycardia unresponsive to vasopressin and other life-supporting procedures. Another patient died of septicemia.

## Discussion

The present study was focused on hemodynamic instabilities and clinical events following surgery for congenital cardiac communications. To investigate the role of Down syndrome and other clinical features in predisposing patients to such instabilities, the PAP/SAP_IPO_ parameter and the behavior of pulmonary and systemic pressure curves were used as primary endpoint. In the present cohort, comprising patients for the majority of whom preoperative cardiac catheterization was not required, peripheral oxygen saturation measured at bedside was found to be the best predictor of postoperative hemodynamics. This was demonstrated by adjusting two different statistical models to the observed data, i.e., the general linear model using a single outcome measure per patient (PAP/SAP_IPO_), and the repeated-measures general linear model using the pulmonary artery pressure curve as outcome. In both instances, preoperative oxygen saturation was analyzed as a covariate in the model. At first sight, Down syndrome and the presence of atrioventricular septal defect seemed to be associated with altered hemodynamics as well. This apparent association was probably due to the fact that the lowest preoperative levels of oxygen saturation were detected in syndromic individuals with atrioventricular canal. Noticeably, however, several syndromic patients, some with atrioventricular septal defect had normal preoperative oxygen saturation and did quite well postoperatively. Thus, within the limits of cardiac anomalies we diagnosed in the present cohort, the presence of Down syndrome itself did not seem to influence postoperative hemodynamics. So, the next question is what lies behind decreased levels of oxygen saturation, which are quite frequent in Down syndrome individuals, that renders patients with unrestrictive cardiac communications at increased risk for postoperative instabilities, i.e., pulmonary hypertension.

The idea that comes first is that decreased oxygen saturation is due to right-to-left intracardiac shunting associated with increased pulmonary vascular resistance. Although pulmonary vascular remodeling sounds like an obvious explanation for altered postoperative hemodynamics in these patients, critical levels of pulmonary vascular resistance associated with advanced pulmonary vasculopathy did not seem to constitute the pathophysiological scenario in the present cohort. In fact, the pulmonary-to-systemic blood flow ratio measured by transthoracic echocardiography in 19 patients with Down syndrome, atrioventricular septal defect and peripheral oxygen saturation lower than 95% was 2.20 (1.80–2.90). Moreover, only mildly elevated pulmonary vascular resistance was observed in patients who underwent preoperative cardiac catheterization. A more complete explanation for the association between preoperative oxygen desaturation and altered postoperative hemodynamics should include abnormalities such as intrapulmonary in addition to intracardiac shunting, ventilation-perfusions mismatch of several causes and endothelial dysfunction with vasoconstriction in addition to vascular cell proliferation with remodeling of pulmonary arteries^17^. In the particular setting of Down syndrome, a number of congenital and acquired airway and pulmonary conditions may contribute to intermittent or sustained hypoxia leading to regional or global pulmonary vasoconstriction aggravated by hemodynamic stress (congenital heart defects), endothelial dysfunction and inflammation^18^. Thus, rather than a simple result of right-to-left intracardiac shunting, systemic oxygen desaturation should be looked on as a consequence of several morbid conditions, many with a potential for persistence even after successful cardiac surgery, serving as a pathophysiological basis for hemodynamic instabilities.

Another point to be addressed in this context, is the biological crosstalk between elements of small airways and vessels in the lungs. These structures are inserted in the indivisible microenvironment of the distal lung in such a way that biological phenomena occurring on one side (airways or vessels) have inevitable effects on the other^19^. Viral respiratory infections, which are very frequent and recurrent in pediatric subjects with congenital cardiac shunts, may be taken as an example of this situation. Respiratory epithelial cells infected by viruses express a number of cytokines, chemokines, growth factors and related molecules that are central to the pathophysiology of bronchiolitis and asthma^20,21^. The majority of such molecules were also shown to play a pivotal role in pulmonary vascular remodeling. For example, chemokine RANTES (regulated on activation, normal T-cell expressed and secreted) is highly expressed during infections by respiratory syncytial virus, rhinovirus and other agents^22,23^, and was shown to be upregulated, along with its receptor, in the endothelium of pulmonary arterial hypertensive patients^24,25^. Rhinovirus was also shown to induce the expression of chemokine IP-10 (interferon-gamma-inducible protein 10) which plays a central role in vascular smooth muscle cell proliferation and pulmonary arterial remodeling^9,10,26^. Thus, airway and vascular remodeling are closely related processes in the lungs. In a recent study of ours, the presence of genetic material for respiratory viruses in tracheal aspirates of pediatric patients undergoing surgery for congenital cardiac shunts was found to be a risk factor for heightened pulmonary arterial pressure postoperatively^27^.

In the present study, serum levels of several inflammatory proteins increased postoperatively compared to baseline, largely reflecting the systemic inflammatory reaction that follows the use of cardiopulmonary bypass, while the levels of other proteins decreased. In two instances, there were reasons for the observed decrease. The level of chemokine RANTES was influenced by the use of inotropic and vasoactive drugs which is generally associated with depletion of T lymphocytes. The decrease in CD40L may be explained, at least in part, by the marked reduction in platelet count, since platelets are a major source of CD40L in circulation. Interestingly, postoperative levels of some inflammatory markers were higher in patients presenting with baseline oxygen saturation lower than 95% compared to those who were at or above this level. Importantly, this was also seen within the group of Down syndrome patients. The possibility exists that mechanisms involving proinflammatory pathways are triggered preoperatively and become exacerbated under perioperative stimuli. The observation of normal baseline levels for almost all inflammatory proteins was not surprising, since determinations were performed in serum, not at tissue level. The duration of cardiopulmonary bypass could not totally explain the observed changes in serum proteins. Because nearly all of the aforementioned proteins were shown to be involved in pulmonary vascular remodeling^28–30^, we speculate that the acute inflammatory storm may also have more prolonged biological effects on pulmonary vessels as to cause persistent hemodynamic alterations late after surgery^31^. Considering the Down syndrome group specifically, the observed associations of baseline oxygen saturation with postoperative inflammation and hemodynamics suggests the existence of a particular subset of high-risk individuals potentially identifiable at preoperative bedside examination.

The study has obvious limitations, some deserving consideration. Our observations cannot be extrapolated to neonates and infants with more complex cardiac anomalies. Although these are extremely important subsets of patients in terms of postoperative complications and management, we felt that their inclusion would increase considerably the complexity of the study, making it difficult to come to simple and useful conclusions. Besides, in view of the limitations of transthoracic echocardiography in providing hemodynamic parameters in some instances, we could not have a complete noninvasive preoperative evaluation in some patients. Right heart catheterization could be the solution but currently, it has restricted indications in this population. So, the hemodynamic profile of our patients was defined by directly measuring pulmonary and systemic arterial pressures in the operating room, before the initiation of cardiopulmonary bypass. Finally, refining diagnosis in Down syndrome patients by including in depth evaluation of structural and functional abnormalities in upper and lower airways would help understanding the pathophysiological mechanisms underlying altered oxygen saturation.

Based on our data, we would like to conclude that the presence of Down syndrome itself does not necessarily constitute a risk factor for postoperative hemodynamic instabilities in patients undergoing surgery for congenital cardiac communications. Rather, decreased oxygen saturation, which is quite frequent in Down syndrome individuals, seems to be the tip of the iceberg over a number of pathophysiological phenomena with potential impact on postoperative hemodynamics. The risk of having high levels of pulmonary artery pressure postoperatively seems to be 5 times greater in patients with a preoperative peripheral oxygen saturation < 95% compared to those who are above this level. Although further studies are required to better understand how initial pathophysiological phenomena leading to altered oxygen saturation influence the inflammatory response to surgery, as suggested by our results, it is reasonable to suppose that acute inflammation acts in combination with preoperative changes in pulmonary microenvironment to determine the abnormal response of the pulmonary (and systemic) circulation postoperatively.

## Data Availability

The data used to support the conclusion of the present study correspond to the projects FAPESP #2015/21587-5 and #2019/18789-5, and are available from the corresponding author upon request.
